# Obturator internus pyomyositis in a young adult: a case report and review of the literature

**DOI:** 10.4076/1757-1626-2-8588

**Published:** 2009-09-03

**Authors:** Dimitrios D Nikolopoulos, Alexandros Apostolopoulos, Ioannis Polyzois, Spyros Liarokapis, Ioannis Michos

**Affiliations:** 4th Orthopaedic Department, General Hospital Asklipieion VoulasV. Paulou 01 Str, 166-73, AthensGreece

## Abstract

**Introduction:**

There has been a recent increase in the incidence of myositis worldwide. To this date, myositis has been described almost exclusively in children and adolescents. In most cases the causative agents are Gram-positive bacteria. When it involves muscles around the hip, other differential diagnoses such as septic arthritis and transient synovitis need to be excluded amongst others.

**Case presentation:**

We present the case of a 16 year old Caucasian male who suffered from pyomyositis of his left obturator internus muscle. He was pyrexial at 41°C with rigors and severe hip pain, whilst range of motion of his left hip was severely limited. Tenderness over the left pubic bone was significant. After clinical examination and relevant laboratory investigations, it was decided to treat him conservatively with intravenous antibiotics. Blood cultures grew *Staphylococcus aureus* resistant to amoxicillin and ampicillin. The patient was discharged fifteen days later. At the time of discharge, he was symptom-free.

**Conclusion:**

This form of pyomyositis is a rare pyogenic infection, which may be difficult to diagnose and can easily be missed. Therefore, physicians should familiarise themselves with this condition and consider it as a possible differential diagnosis in patients presenting with an acutely painful hip.

## Introduction

Patients who present with pyrexia and an acutely painful hip require an urgent orthopaedic opinion. Early exclusion of bacterial septic arthritis is essential, as the consequences of delay may prove potentially devastating for the joint. If evaluation rules out the presence of septic arthritis, other conditions such as toxic synovitis, osteomyelitis, discitis, pyomyositis, juvenile rheumatoid arthritis and malignancy should be considered.

Pyomyositis is a primary bacterial infection of skeletal muscle. Initially, it was considered to be a disease of the tropics. However, pyomyositis has become more frequent among both children and adults in temperate climates as well [1]. Although it predominantly affects muscles of the lower limb, it may also involve muscles of the upper limb, trunk and spine. In the lower limb, the most commonly affected muscles are the quadriceps and iliopsoas followed by the gluteal muscles [2]. More rarely, it can involve the obturator internus (OIM) and/or externus muscles [3]. Lack of familiarity with the condition frequently leads to a delayed diagnosis [3,4]. We report herein a case of a 16 year old male with pyomyositis of the OIM.

## Case presentation

A 16-year-old Caucasian male presented to our emergency department complaining of acute onset of left hip pain and was unable to weight bear. He had been pyrexial at 37.6°C for the last 2 days. He described the pain as sharp and constant, located in the left groin and radiating down to the anterolateral aspect of his ipsilateral thigh. It was aggravated by movement. There were no associated abdominal or urinary symptoms. There was no history of trauma, recent illness or foreign travel. There was no relevant past medical history of any note.

On clinical examination, the patient was pyrexial at 38.9°C. His position of comfort was with the hip in flexion, abduction and external rotation. Abdominal examination revealed tenderness throughout the lower quadrants. There were no signs of rebound tenderness and bowel sounds were present. There was some discomfort in the left groin on deep palpation and tenderness in the left buttock lateral to the ischial tuberosity. No inguinal lymph nodes were palpable. The left hip and thigh were tender to palpation, but no erythema or swelling was noted. Movements of his left hip were limited. Specifically, he had an extension of 15°, flexion of 60°, abduction of 15°, adduction of 10°, whilst internal rotation and external rotation were 10° each. Active straight leg-raising was only possible up to 10° and painful. Pace’s and Freiberg’s signs were both positive. No sensory or motor deficit of his left lower limb was noted. All the other joints had a full range of motion and were symptom-free.

Initial laboratory studies revealed a white blood cell count of 11.0 K/uL (neutrophils 90%), CRP 168 mg/l, and ESR 32 mm/h. His serum electrolytes and coagulation screen were within normal limits. Routine radiographs of both hips and pelvis were normal. Urine culture showed no growth. Blood cultures were also performed. Abdominal ultrasound did not reveal any pathology. Subsequently, the patient was admitted to hospital for comprehensive diagnostic workup, bed rest and analgesia. Intravenous antibiotics [ciprofloxacin (400 mg; 1 × 2,) clindamycin (600 mg; 1 × 3) and meropenem (1 gr; 1 × 3)] were commenced empirically after blood cultures were taken. The differential diagnosis included septic arthritis, juvenile rheumatoid arthritis, osteomyelitis with periosteal involvement of the left pubic bone and/or the ischial tuberosity and iliopsoas pyomyositis.

The next day his clinical condition and his laboratory investigations showed considerable deterioration. He was pyrexial at 41° C with rigors and severe hip pain, whilst range of motion of his left hip became severely limited. Tenderness over the left pubic bone increased significantly. His CRP rose to 172 mg/l and the ESR was 46 mm/h. Furthermore, SCOT was 125 U/L, SGPT was 117 U/L and γGT was 46 U/L. The Ra test, ANA, P-ANCA, C-ANCA, Parvo-virus, Cocxackie and Echovirus IgM and IgG were all negative. A bone scintiscan and an MRI of the pelvic/hip and the lumbar spine were performed the following day.

**Figure 1.**
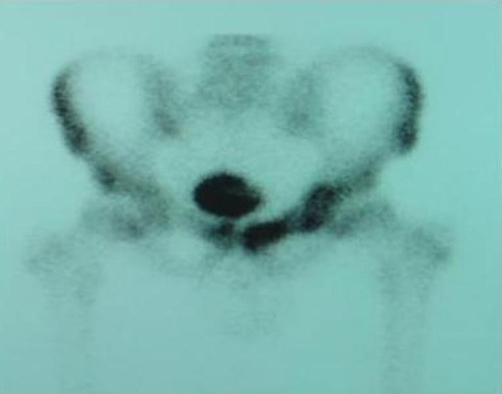
The 3rd phase of 99 mTc ST-scan revealed local increased activity in the region of the left ischium and pubis and the ipsilateral acetabulum, which is associated with osteomyelitis of these regions.

**Figure 2.**
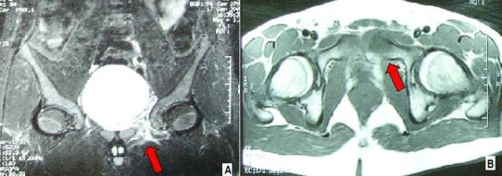
**(A, B)** Pelvic/hip MRI demonstrating slightly increased signal in the left pubis, adjacent to the pubic symphysis, suggesting ostietis pubis or osteomyelitis. Increased signal reveals mainly to the proximal segment of the obturator internus muscle and hereupon to the obturator externus muscle with little fluid collection, and slight edema of the soft tissue, but with no abscess cavities.

The 99 mTc CT-scan revealed increased local activity in the region of the left ischium, pubis and the ipsilateral acetabulum at all three phases. These findings were in line with osteomyelitis (Figure 1). The MRI of his lumbar spine was unremarkable, whereas the pelvic/hip MRI demonstrated slightly increased signal in the left pubis, adjacent to the pubic symphysis suggesting osteitis pubis, although osteomyelitis could not be excluded. Increased signal was revealed also in the proximal segment of the obturator internus muscle and the obturator externus muscle with minimal fluid collection and slight edema of the neighboring soft tissues. No abscess cavity was seen (Figure 2). Meanwhile blood cultures grew *Staphylococcus aureus* resistant to amoxicillin and ampicillin but sensitive to ciprofloxacin, clindamycin and teicoplanin. After consultation with the department of microbiology, it was decided to replace meropenem with teicoplanin (400 mg; 1 × 1 IV). This regime carried on for three weeks.

Seventy-two hours after the initiation of teicoplanin the patient became apyrexial and his local symptoms subsided. A week later his gait returned to normal. There was no more tenderness over the left pubic bone. His ESR dropped to 24 mm/h. The patient was discharged fifteen days later. At the time of discharge, he was symptom-free. He carried on with oral antibiotics (ciprofloxacin 500 mg; 1 × 2 per os) for one month. On his last follow-up at six months in the outpatients department, he was completely asymptomatic and had returned to his previous normal daily activities. His ESR and CRP were within normal limits.

## Discussion

The obturator internus muscle originates from the anterolateral wall of the pelvic cavity around the obturator foramen. It exits the pelvic cavity through the lesser sciatic foramen and inserts into the greater trochanter along with the superior and inferior gemelli. It abducts and externally rotates the hip joint [1].

Pyomyositis is a primary, subacute, deep bacterial infection of the skeletal muscle that manifests itself as localized abscess formation, but may also present as a diffuse inflammatory or a rapidly progressing myonecrotic process [5,6]. The disease is common in many parts of Africa and the South Pacific, thus the name “tropical” pyomyositis. During the past decades its incidence has been increasing in non-tropical areas [6]. Because of its rarity and often vague clinical presentation, it is unlikely to be considered during the initial differential diagnosis. Moreover, the diagnosis may be delayed as the affected muscle is deeply situated and local signs are not apparent. This delay in diagnosis may result in increased morbidity and sometimes a significant mortality rate [5]. It may result in compartment syndrome, septic arthritis with serious complications and very rarely septicemia resulting in death [5,6]. Lung and brain abscesses, pericarditis, myocarditis, endocarditis and renal failure have also been reported. The long-term sequelae of pyomyositis include osteomyelitis of adjacent bones, muscle-scarring, residual weakness and functional impairment [5].

The most frequently affected muscles are the quadriceps, hamstrings, gluteus and iliopsoas [1,6]. Pyomyositis originating in the obturator internus or/and externus muscles is exceptionally rare. As a whole, it occurs most commonly in children and young adults whilst it exhibits a 2:1 prevalence in male patients [4-6]. Nevertheless, obturator internus or/and externus pyomyositis is more common in females [7]. One possible explanation is the close proximity of the obturator internus muscle to the female reproductive organs [4,7,8].

The usual pathogens are *Staphylococcus aureus* and *Streptococcus pyogenes*. In a North American study, blood cultures were positive in 31% cases of pyomyositis. In this study, purulent material obtained by needle aspiration was positive for *S. aureus* in 70% of all cases and 90% in pediatric patients [9]. Other pathogens described in the literature are *Escherichia coli*, *Haemophilus influenzae*, *Citrobacter freundii*, *Fusobacterium*, *Peptostreptococcus*, *Neisseris gonorrhoeae*, *Klebsiella pneumonia*,* Serratia marcescens* and *Yersinia enterocolitica* [2,4,5,10]. Infection may spread to the obturator internus muscle directly from trauma, from the gut or via the female reproductive tract [8,9].

Local trauma is a recognized initiating factor for pyomyositis [1,5] and has been documented between 21% to 66% of all cases [1,5,11] Some cases have been attributed to adjacent osteomyelitis or to more superficial skin infections. A potential source of bacteraemia is also frequently identified, such as eczema of the feet as reported by King et al. [11]. It has been suggested that primary myositis begins when muscle haematoma from a recent injury becomes colonized during an episode of bacteraemia [4,6,11]. In tropical cases, malnutrition, scurvy, thiamine deficiency, as well as certain infections, notably Dracunculus, malaria, filariasis, arbovirus, and leptspirosis seem to increase the risk of pyomyositis. Other associated conditions in temperate cases include HIV, via multiple proposed mechanisms; diabetes mellitus, leukemia, asplenia, SLE, scleroderma, Felty’s, zoster, measles, picornavirus, coxsackie, arenavirus, sickle cell disease, cancer chemotherapy, alcoholism, decubitus ulcers, osteomyelitis and aplastic anemia [5].

The clinical presentation of obturator internus muscle pyomyositis resembles that of septic arthritis of the hip or osteomyelitis [5]. In a child who presents with fever, limp, and hip pain, septic arthritis of the hip must be suspected and excluded. If septic arthritis is not confirmed, other conditions such as toxic synovitis, osteomyelitis, discitis, pyomyositis, juvenile rheumatoid arthritis, and malignancy should be considered [1-3,6,8]. The most common physical findings are restriction of all hip movements and tenderness over the hip and groin region [1,3,4,6]. An enlarging obturator internus abscess may cause pressure on the obturator or sciatic nerve thereby causing radiating leg pain (Table 1) [1,2,6]. As the obturator internus muscle is deeply located, infection can be difficult to diagnose clinically.

**Table 1.** Clinical stages of pyomyositis

Stage 1	- Crampy local muscle pain, swelling, low-grade fever, mild leukocytosis, induration of muscles leading to a ‘woody’ texture.
	- No fluctuance and aspiration with not yield pus.
	- About 2% of patients present in this stage.
Stage 2	- 10-21 days after onset of symptoms.
	- Fever, exquisite tenderness, edema, marked leukocytosis with eosinophilia in many tropical cases.
	- Aspiration will yield pus.
	- > 90% cases present at this stage.
Stage 3	- Bacteremia, toxic appearance, marked fluctuance.

Furthermore, laboratory studies for pyomyositis are not very specific. Early radiological evaluation is essential. The diagnosis always requires MRI confirmation nowadays, whereas sometimes 99 mTc ST-scan is very helpful. Computed tomography may also be used to guide pus aspiration [11]. MRI appears to be the most sensitive test [5,10-13].

For our young male patient in this case, it is important firstly to mention the acute onset of painful symptoms from his hip in association with a pyrexia of 41° C and rigors. Of note, this patient did not have a relevant past medical history (trauma, recent illness or foreign travel). The second notable point is the considerable deterioration of his clinical condition after 24 hours. His pain grew in severity, whilst the range of motion of his left hip became severely limited. His position of comfort was with the hip in flexion, abduction and external rotation. With this clinical image we considered the possibility of septic arthritis. Nevertheless, our differential diagnosis also included juvenile rheumatoid arthritis, osteomyelitis with periosteal involvement of the left pubic bone and/or the ischial tuberosity and iliopsoas pyomyositis.

The fact that there was some discomfort over his left groin on deep palpation and the associated significant tenderness over his left pubic bone led us to thoroughly investigate the possibility of hip pyomyositis. The MRI and the 99 mTc ST-scans that were performed were extremely helpful in demonstrating the diagnosis of a rare case of obturator internus pyomyositis. As it has already been mentioned, the long-term sequelae of pyomyositis include osteomyelitis of adjacent bones, muscle-scarring, residual weakness and functional impairment [5]. In our case the 99 mTc ST-scan demonstrated osteomyelitis of the left pubis (Figure 1). The thorough clinical examination and laboratory investigations in association with the results of the blood cultures that grew* Staphylococcus aureus* resistant to amoxicillin and ampicillin helped us to decide in favour of a conservative treatment with intravenous antibiotics. Consequently, his clinical condition improved rapidly without the need for surgical drainage. More importantly, the patient recovered completely without any long-term sequelae.

In most of the patients with obturator internus pyomyositis, treatment with intravenous antibiotics alone is sufficient. Antibiotics of choice should be efficient against *S. aureus*, for instance nafcillin or cefazolin [5]. Blind coverage for immunocompromised patients should include broader spectrum coverage for gram negatives and anaerobic organisms, such as gentamine and clindamine [5,8,9,12]. Drainage is indicated where pus is present. The duration of antibiotics has not been established and can vary from 2 to 6 weeks, depending on the clinical severity and patient response to antibiotics [5,8,9,12]. Most individuals recover completely with no long-term sequelae.

## Conclusion

We present a rare case of obturator internus pyomyositis in a 16-year-old Caucasian male and we highlight the importance of clinical examination and laboratory tests in association with the relevant radiological investigations in the form of MRI and 99 mTc ST-scans. A complete systematic clinical examination, including examination of the abdomen and groin in every child or young adult that presents with a painful hip and pyrexia, is mandatory and was extremely helpful in establishing our final diagnosis.

Obturator internus muscle infection should be included in the differential diagnosis of a child or adolescent presenting with fever, limp and hip/abdominal pain. Early and accurate diagnosis is important as the condition is curable. Otherwise, infection may extend to adjacent structures and if uncontrolled, may cause permanent disability or even death in extreme cases. Early diagnosis, established with MRI or CT, prevents surgical intervention and allows successful treatment with antibiotics. When pyomyositis of the OIM is diagnosed, an antistaphylococcal antibiotic such as semi-synthetic penicillin or a second-generation cephalosporin should be initiated. Surgical drainage is indicated if the patient fails to respond to medical therapy or if an unusual organism is suspected, as is commonly seen in immunocompromised patients.

## Abbreviation

OIM: obturator internus muscle
